# Creation and Piloting of a Model for Simulating a Minimally Invasive Myomectomy

**DOI:** 10.7759/cureus.4223

**Published:** 2019-03-11

**Authors:** Mary N Towner, Yolianne Lozada-Capriles, Amy LaLonde, Ashkan Ertefaie, Jonathan Stone, Bala Bhagavath, Ahmed Ghazi

**Affiliations:** 1 Obstetrics and Gynecology, University of Rochester, Rochester, USA; 2 Biostatistics and Computational Biology, University of Rochester, Rochester, USA; 3 Neurosurgery, University of Rochester, Rochester, USA; 4 Urology, University of Rochester, Rochester, USA

**Keywords:** laparoscopic myomectomy, robotic, myomectomy, resident education, minimally invasive surgery, surgical simulation, fibroids

## Abstract

Introduction: In the era of mandatory work hour restrictions for residency programs, the opportunity for mastery of complex surgical skills in the operating room (OR) has been compromised. All the while, gynecologic surgical techniques have continued to expand. Surgical simulation offers an adjuvant modality for helping young surgeons hone their surgical techniques. We sought to design, construct, and pilot a model for simulating a minimally invasive myomectomy procedure for the purpose of resident training. We undertook a preliminary evaluation of the model’s validity.

Methods: Gynecologic surgical simulation models were constructed from polyvinyl alcohol poured into 3D-printed injection molds. A total of 12 laparoscopic and 12 robot-assisted simulated myomectomies were performed using the models. Face and content validity were evaluated with post-simulation questionnaires. Construct validity was assessed by comparing procedural metrics (time to completion and estimated blood loss) between residents and attending surgeons.

Results: In the post-simulation survey, the majority of attending surgeons agreed the model was realistic (83.3%) and included the critical steps of a myomectomy (87.5%). Most residents agreed they would feel more prepared for a myomectomy if they practiced on the model beforehand (87.5%) and the majority of attending surgeons agreed they would feel comfortable giving a resident more operative autonomy if the resident had previously completed the simulation (71.4%). Procedural metrics were not significantly associated with expertise level.

Conclusion: We were able to successfully create a model for simulating a minimally invasive myomectomy. Initial simulations using the model were well received by participants. Further development and investigation of the model will be pursued to determine if this is a valid and useful tool for teaching and practicing a minimally invasive myomectomy.

## Introduction

The training of obstetrics and gynecology residents has struggled to keep up with the pace of surgical advancement. Just as entirely new approaches to hysterectomy, myomectomy, and other procedures were becoming ubiquitous in gynecologic surgery, residents began spending less time in the operating room. Work hour restrictions enforced since 2003, which in some programs decreased weekly work hours by nearly 25%, have reduced the number of surgical procedures performed by residents [1]. Meanwhile, residents are expected to become proficient in an expanding array of surgical procedures. This conflict has likely contributed to reports that over half of new obstetrics and gynecology residency graduates are considered unable to independently perform basic gynecologic surgeries, such as abdominal hysterectomy or hysteroscopy, while less than one-quarter of new graduates are capable of performing a vaginal hysterectomy on their own [2].

Fortunately, as technologic advances present new surgical techniques to learn, they also introduce creative platforms for instructing young surgeons. Surgical simulation continues to garner significant interest as a possible solution to the predicament of training surgical residents in the 21st century. Though most studies on the topic are relatively small, simulation has been found to improve overall operative performance, in both simulated and live surgery [3]. Several systematic reviews have found residents who participate in the surgical simulation, particularly proficiency-based programs, perform better in the operating room (OR) [4]. Current simulators, however, are often unrealistic or exceedingly expensive and the majority of commercially available simulators have not been formally validated [5]. We describe below the creation of a life-like simulation model to practice minimally invasive myomectomy. In this pilot study, we sought to evaluate the model and its utility as a tool for resident surgical education.

## Materials and methods

**Figure 1:**
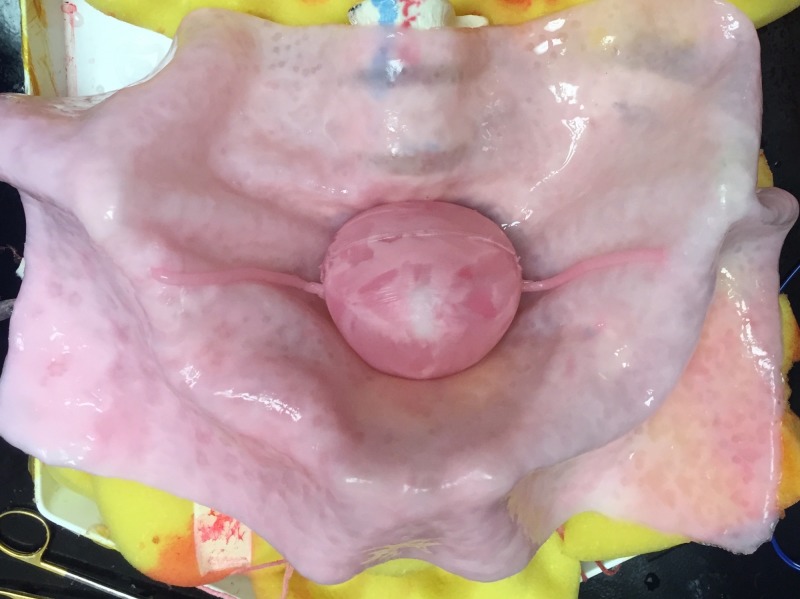
Model prior to placement in the laparoscopic box trainer

Models were created using a technique developed at our institution, previously applied to non-gynecologic surgical simulation models [6]. A computer-aided design model of each anatomic component (uterus, fallopian tube, ovary, myoma, and blood vessel) was constructed using Meshmixer, version 11.0 (Autodesk, San Rafael, CA, USA) in consultation with a gynecologic surgeon who performs a high volume of robot-assisted myomectomies at our institution. Molds were printed using a 3D printer (Cube X, 3D Systems, USA), then injected with polyvinyl alcohol, which has been used with success in previous studies to create surgical simulation models. The molds were then subjected to several freeze-thaw cycles. Each model had one 3.5 cm anterior myoma. Bleeding of the myoma was achieved by perfusing a network of vessels embedded into the model with a bag of artificial blood (red-dyed saline), hung to approximately the same height during each simulation to ensure consistent pressure. The model was placed into an artificial pelvis, which was then secured in a laparoscopic training box (Figure 1).

**Figure 2:**
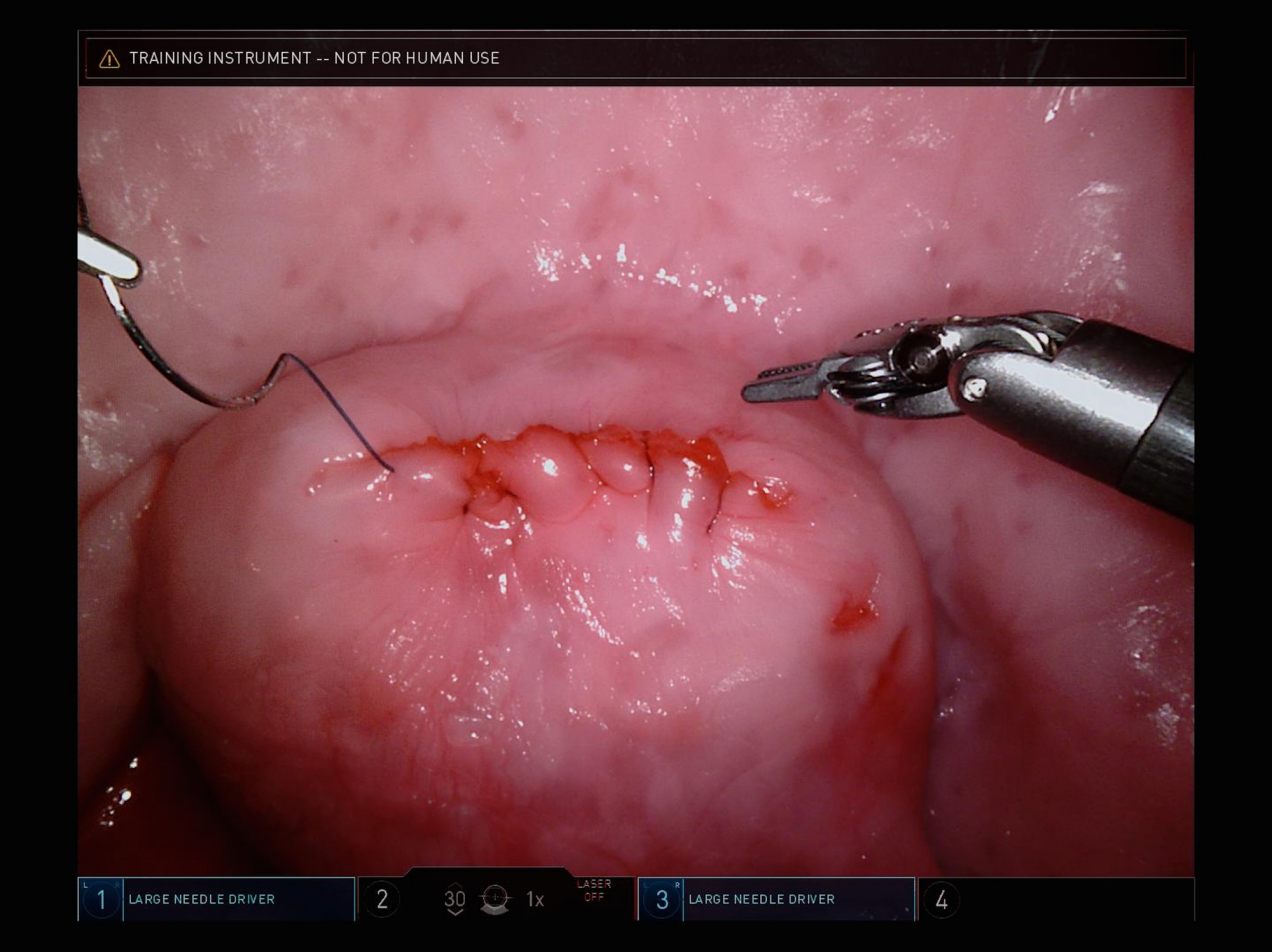
Simulation model as seen through the robotic laparoscopic camera

For the laparoscopic myomectomy, a 5-mm laparoscope was inserted into the laparoscopic training box and a set of standard laparoscopic instruments were provided. For the robot-assisted myomectomy, the da Vinci Xi surgical system (Intuitive Surgical Inc., Sunnyvale, CA) was docked to the training box. Figure 2 represents an example image of the model as seen during robotic simulation. 

Our university’s Institutional Review Board granted approval for the study. From March 2016 through March 2017, third- or fourth- year obstetrics and gynecology residents, minimally invasive gynecologic surgery (MIGS) fellows, female pelvic medicine and reconstructive surgery (FPMRS) fellows, and attendings were recruited. While all residents had prior experience with minimally invasive myomectomies, there was no required minimum number of procedures. Only attending surgeons credentialed to perform laparoscopic or robot-assisted procedures were approached. As fellows had already completed residency, they were included in the attending group. Study participants were assigned to a laparoscopic or robot-assisted procedure based on OR and participant availability, with the intent to complete a balanced number of laparoscopic and robotic simulations. If an attending surgeon was not credentialed in robot-assisted surgery, they were assigned to laparoscopy. Four study participants volunteered to participate in both a laparoscopic and robot-assisted simulation, which was permitted.

Laparoscopic simulations took place in our simulation center - a large, private room with several laparoscopic training stations. Robot-assisted simulations took place in robotic ORs. Study participants provided verbal informed consent prior to participation. Study participants were advised that the procedure would be recorded, and videos would be saved anonymously and without audio. Study participants were informed of the quantitative metrics to be documented, including time to completion (time from initiation of dissection to cutting suture after repair of the defect), estimated blood loss (EBL), as calculated by the amount of artificial blood lost from bag, and each participant’s number of years of surgical experience. Each participant was instructed to remove the myoma and repair the uterus using barbed suture in a continuous fashion and in a single layer, due to limitations in the material’s strength.

**Figure 3:**
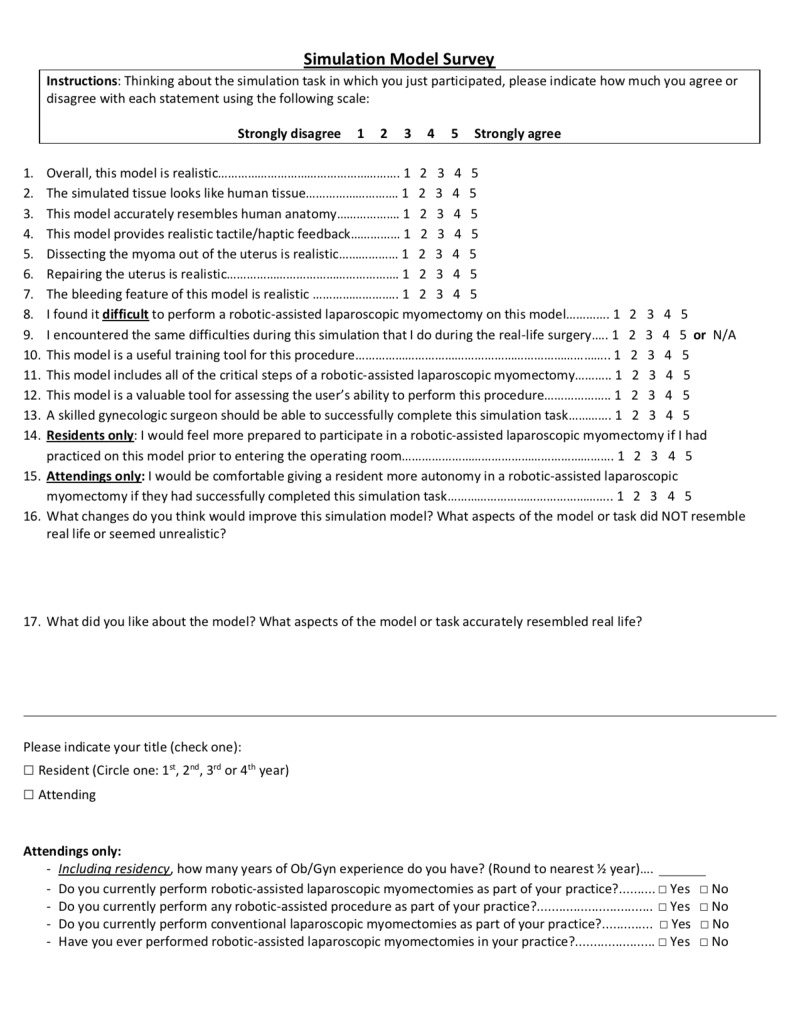
Post-simulation survey filled out by study participants

Immediately after completing the simulation, study participants completed a survey using a visual analog scale (VAS), where 1 always represented the most negative response and 5, the most positive (Figure 3).

This survey was intended to assess both the aesthetic and functional realism of the model (face validity), as well as its utility for practicing uterine myomectomies (content validity). As no validated survey for myomectomy simulation has been described, we used surveys from other simulation studies to guide the creation of our own, which was then reviewed by two authors with experience in surgical simulation and the high-volume gynecologic surgeon who assisted in the design of the model [7]. After the initial three simulations, the survey was expanded to include six additional statements to better define which aspects of the model most impacted its utility as a surgical learning tool.

Study participants’ survey responses and quantitative data were saved anonymously on a secure university-licensed Box.com (Redwood City, CA, USA) account. Statistical analysis was performed using SAS software (Cary, NC, USA). If any participant left a survey statement blank, they were removed from analysis for that statement. Survey responses and procedural metrics from study participants who completed both simulations were treated as independent data. Face and content validity were evaluated by calculating the percentage of study participants who responded positively to survey statements (defined as a VAS score of 4-5). Wilcoxon rank sum tests were performed to address comparisons of procedural metrics, as well as median VAS scores, between both residents and attendings, as well as between the laparoscopic and robotic groups.

## Results

Twenty volunteers participated in 12 laparoscopic and 12 robot-assisted simulations. There were 16 resident simulations, divided evenly between laparoscopic and robot-assisted. Eight residents were in their third year of training and eight were in their fourth year. There were eight attending simulation sessions, again divided evenly between laparoscopic and robot-assisted. Attendings had an average of 8.5 years’ experience, with a range of 5-19 years. Two fellows participated in the study; one MIGS fellow performed a laparoscopic simulation and one FPMRS fellow performed a robot-assisted simulation. The robotic surgical system allows for recording of surgical procedures; a compilation of these recordings can be seen in Video 1. 

**Video 1:** Compilation of footage from robotic myomectomy simulation sessions

Out of 24 simulations, 23 were successfully completed. The one participant who was unable to complete the task was a third-year resident. The average time to completion was 34.16 (±11.76) minutes for conventional laparoscopic myomectomy and 25.07 (±10.27) minutes for robot-assisted myomectomy. The estimated blood loss was 109.4 (±95.37) milliliters for conventional laparoscopy and 56.25 (±32.04) milliliters for robot-assisted myomectomy. These differences were not statistically significant. Procedural metrics based on the level of expertise can be seen in Table 1. As between surgical modality groups, there was no statistically significant difference between procedural metrics in the resident and attending groups.

**Table 1:** Procedural metrics, by training level Data presented as mean (standard deviation)

	Attendings	Residents	p Value
Time to completion (minutes)	30.7 (±12.4) [n=6]	29.5 (±11.9) [n=15]	0.788
Estimated blood loss (milliliters)	56.2 (±31.4) [n=4]	91.7 (±82.8) [n=12]	0.626

A summary of the median VAS scores for each post-simulation survey statement, categorized by level of training and mode of simulation, are shown in Tables 2-3, respectively. Because some participants failed to respond to every statement and because the survey was expanded after the first three simulations, not every statement received 24 responses. There were no statistically significant differences in VAS scores between participants who completed a laparoscopic myomectomy compared to the robot-assisted myomectomy. In general, resident and attending surgeon responses to survey statements were comparable, though attendings scored the surgical (tactile) feedback offered by the model significantly more highly than residents; this was the only statistically significant difference between resident and attending VAS scores (4 vs 3, p=0.035).

**Table 2:** Median visual analog scale (VAS) score for post-simulation survey statements, by level of training VAS = visual analog scale, IQR = interquartile range

	Overall [n=24 unless otherwise noted]	Resident [n=16 unless otherwise noted]	Attending [n=8 unless otherwise noted]	p Value
Model is realistic	4 (IQR 4-5) [n=22]	4 (IQR 3.5-4.5)	4 (IQR 4-5) [n=6]	.682
Looks like human tissue	4 (IQR 4-5)	4 (IQR 4-5)	4 (IQR 4-4.5)	.728
Accurately resembles human anatomy	5 (IQR 4-5)	5 (IQR 4-5)	4.5 (IQR 4-5)	.667
Provides realistic surgical feedback	4 (IQR 3-4)	3 (IQR 3-4)	4 (IQR 4-4.5)	.035
Dissection of myoma is realistic	4 (IQR 3-4) [n=22]	4 (IQR 2.5-4)	4 [6]	.484
Repairing uterus is realistic	3.5 (IQR 3-4) [n=22]	3 (IQR 3-4.5)	4 (IQR 3-4) [n=6]	>0.99
Bleeding feature is realistic	4.5 (IQR 4-5) [n=14]	5 (IQR 4-5) [n=11]	4 (IQR 2-4) [n=3]	.116
Found it difficult to perform procedure	3 (IQR 1.5-4)	3 (IQR 1.5-4)	3 (IQR 1.5-4)	.711
Encountered same difficulties on model as in live procedure	4 (IQR 3-5) [n=20]	4 (IQR 3-5) [n=13]	4 (IQR 3-4) [n=7]	.719
Model is a useful training tool	5 (IQR 4-5) [n=22]	5 (IQR 4-5)	4.5 (IQR 4-5) [n=6]	.741
Includes all of the critical steps of the procedure	5 (IQR 4-5)	5 (IQR 4.5-5)	4.5 (IQR 4-5)	.342
Model is valuable tool for assessing user’s ability	4 (IQR 3-5)	4 (IQR 3-5)	4.5 (IQR 3.5-5)	.667
Skilled gynecologic surgeon should be able to successfully complete simulation	5 (IQR 4-5)	5 (IQR 4-5)	5 (IQR 4.5-5)	.904
Would feel more prepared to participate in live surgery after practicing on this model (residents only)	5 (IQR 4-5) [n=16]	5 (IQR 4-5)	-	-
Would feel comfortable giving more autonomy to resident if they had completed this simulation (attendings only)	5 (IQR 3-5) [n=7]	-	5 (IQR 3-5) [n=7]	-

**Table 3:** Median visual analog scale (VAS) score for post-simulation survey statements, by mode of simulation VAS = visual analog scale, IQR = interquartile range *p value not calculated due to n<5 in both groups

	Overall [n=24 unless otherwise noted]	Conventional laparoscopy [n=12 unless otherwise noted]	Robot-assisted [n=12 unless otherwise noted]	p Value
Model is realistic	4 (IQR 4-5) [n=22]	4 (IQR 4-4.5)	4 (IQR 3-5) [n=10]	.841
Looks like human tissue	4 (IQR 4-5)	4 (IQR 4-5)	4 (IQR 4-5)	.772
Accurately resembles human anatomy	5 (IQR 4-5)	5 (IQR 4-5)	4 (IQR 4-5)	.258
Provides realistic surgical feedback	4 (IQR 3-4)	4 (IQR 2.5-4)	4 (IQR 3-4)	.904
Dissection of myoma is realistic	4 (IQR 3-4) [n=22]	4 (IQR 3-4)	3.5 (IQR 3-4) [n=10]	.976
Repairing uterus is realistic	3.5 (IQR 3-4) [n=22]	4 (IQR 3-4)	3 (IQR 2-4) [n=10]	.139
Bleeding feature is realistic	4.5 (IQR 4-5) [n=14]	5 (IQR 3-5) [n=7]	4 (IQR 4-5) [n=7]	>0.99
Found it difficult to perform procedure	3 (IQR 1.5-4)	4 (IQR 2-4)	2 (IQR 1.5-3)	.073
Encountered same difficulties on model as in live procedure	4 (IQR 3-5) [n=20]	4 (IQR 3-5)	3 (IQR 3-4.5) [n=8]	.787
Model is a useful training tool	5 (IQR 4-5) [n=22]	4.5 (IQR 4-5)	5 (IQR 4-5) [n=10]	.764
Includes all of the critical steps of the procedure	5 (IQR 4-5)	5 (IQR 4.5-5)	5 (IQR 4-5)	.401
Model is valuable tool for assessing user’s ability	4 (IQR 3-5)	4.5 (IQR 2.5-5)	4 (IQR 3.5-5)	.795
Skilled gynecologic surgeon should be able to successfully complete simulation	5 (IQR 4-5)	5 (IQR 4-5)	5	.358
Would feel more prepared to participate in live surgery after practicing on this model (residents only)	5 (IQR 4-5) [n=16]	4 (IQR 4-5) [n=8]	5 (IQR 4.5-5) [n=8]	.230
Would feel comfortable giving more autonomy to resident if they had completed this simulation (attendings only)	5 (IQR 3-5) [n=7]	5 (IQR 3-5) [n=3]	4.5 (IQR 3.5-5) [n=4]	*

The percentage of positive responses to survey statements, categorized by level of expertise, can be seen in Table 4. The majority of both resident and attending surgeons found the model to be realistic (75% and 83%, respectively). Additionally, 92% of all participants thought the model looked like human tissue and 96% agreed the model accurately represented human anatomy. For dissection of the myoma, 64% of all subjects found the model to be realistic, while 45% said the same of the uterine repair. Among attendings, 88% felt the model included the critical steps of a myomectomy. Importantly, 94% of residents agreed the model was a useful tool for practicing uterine myomectomy. 

**Table 4:** Percentage positive responses (agree or strongly agree) to survey statements CI = 95% confidence interval *One-sided 97.5% confidence interval

	Residents [n=16 unless otherwise noted]	Attendings [n=8 unless otherwise noted]
Model is realistic	75 (CI 8, 93)	83 (CI 36, 100) [n=6]
Looks like human tissue	87 (CI 62, 98)	100 (CI 63*)
Accurately resembles human anatomy	100 (CI 79*)	87 (CI 47, 100)
Provides realistic surgical feedback	44 (CI 20, 70)	100 (CI 63, 100)
Dissection of myoma is realistic	56 (CI 30, 80)	83 (CI 36, 100) [n=6]
Repairing uterus is realistic	44 (CI 20, 70)	67 (CI 22, 96) [n=6]
Bleeding feature is realistic	82 (CI 48, 98) [n=11]	67 (CI 9, 99) [n=3]
Found it difficult to perform procedure	31 (CI 11, 59)	37 (CI 8, 75)
Encountered same difficulties on model as in live procedure	61 (CI 32, 86) [n=13]	57 (CI 18, 90) [n=7]
Model is a useful training tool	94 (CI 70, 100)	83 (CI 36, 100) [n=6]
Includes all of the critical steps of the procedure	94 (CI 70, 100)	87 (CI 47, 99)
Model is valuable tool for assessing user’s ability	62 (CI 35, 85)	75 (CI 35, 97)
Skilled gynecologic surgeon should be able to successfully complete simulation	94 (CI 70, 100)	87 (CI 47, 99)
Would feel more prepared to participate in live surgery after practicing on this model (residents only)	87 (CI 62, 98)	-
Would feel comfortable giving more autonomy to resident if they had completed this simulation (attendings only)	-	71.4 (CI 29, 96) [n=7]

The bleeding feature of the model functioned as intended in 67% (n=16/24) of simulation sessions. There was no significant difference in VAS scores of face validity between respondents whose model bled compared to those whose model did not. However, compared to non-bleeding models, those that bled received significantly higher median VAS scores for the question of whether the simulation included all of the critical steps of the procedure (4 vs 5, p=0.01).

## Discussion

In this initial pilot study, assessment of our model’s face validity was promising. While the majority of the respondents were residents, who would not be considered experts in minimally invasive myomectomies, these senior residents had experience performing a variety of gynecologic procedures. We believe this confers the ability to assess the face validity of a female pelvic simulation model.

Findings regarding the model’s content validity were also encouraging, with the majority of subjects agreeing the simulation included the key surgical steps and would enhance their comfort performing the procedure (residents) or allowing a trainee to do so (attendings) in the OR. One participant remarked, “finding the plane of dissection is the most challenging part for (the real surgery) and (the model) accurately represented that.” Participants also noted the model is “useful for practicing fine skills, movements, and suturing” and described it as a “very useful teaching tool.”

It is interesting to note that attending surgeons rated the tactile feedback significantly more positively than the resident survey participants. We postulate that this may be due to the fact that attending surgeons have simply operated on a wider variety of tissue types and are better able to adapt and respond to a tissue that is not stereotypical. As such, the slightly more rigid tissue of our model did not negatively impact their perception of the model's haptic feedback.

Many participants offered suggestions for improvement. A recurrent comment mentioned the model’s material, which sometimes tore during the uterine repair - likely why this step received low VAS scores. For future models, we have altered the concentration of polyvinyl alcohol and number of freeze-thaw cycles to enhance the material’s strength. Our results also suggest the bleeding feature meaningfully enhances the content validity of the model. We have modified our design to prevent the blood vessel from becoming kinked, which was a major reason for failure of the bleeding feature.

The appraisal of the model’s construct validity was less positive, with residents and attending surgeons requiring no significant difference in time to complete the task. Though differences in EBL were likewise not statistically significant, residents did have 1.5 times greater blood loss compared to attendings. It is likely the lack of significance in these results is at least partially due to the small sample size. However, it should be noted that the difference in years of experience between residents and attending surgeons was, in some cases, only 1-2 years. As such, distinctions in surgical ability may be too subtle to yield differences in performance as measured in our investigation. In future studies, we plan to incorporate the number of surgical cases completed into collected demographic data as a more robust method of stratifying participants’ expertise level.

A major limitation of our study is the small sample size. Recruitment was primarily limited by the number of eligible participants at our institution and the fact that models were built by one resident researcher, limiting the frequency with which simulations could be performed. In addition, each simulation was coordinated by the same resident; arranging simulation sessions mutually convenient for the volunteers and resident researcher proved challenging.

Another limitation of our study is that, in order to maximize participation and evaluate the model in different settings, we simulated both laparoscopic and robotic modalities. This presents a confounding effect. However, as there was no significant difference in VAS scores between the laparoscopic and robotic groups, this did not apparently have a measurable impact on our findings.

Another potential confounding factor is that participants were allowed to complete both simulations in order to gain as much feedback as possible. Of the 4 who did so, 3 performed the laparoscopic simulation first. It is possible experiencing the model in one setting affected how the volunteer performed or responded to the survey in the second. Furthermore, the fact that participants volunteered presents a selection bias; those more comfortable performing a minimally invasive myomectomy might be more likely to volunteer. Lastly, participants were assigned their simulation modality based off scheduling permissibility, rather than randomization, which also presents potential unconscious bias.

Ongoing work includes modifying the model to incorporate participant suggestions, as well as to facilitate simulation of a minimally-invasive hysterectomy. Such a model will be subjected to a rigorous assessment of validity. Ideally, this would include an investigation into whether or not performance on the model correlates with performance during the surgery it simulates. As such, one future goal of this project includes assessing whether practicing on our model improves resident performance in the OR. With future studies, a larger sample size will be sought, participants will be randomized to only one mode of simulation, and more demographic data, including operative experience, will be collected.

## Conclusions

As surgical technology evolves, so too must the training of future surgeons. Surgical simulation allows residents to practice surgery in a low-stress environment and has been shown to improve both technical and non-technical surgical skills. The ability to practice a surgical procedure in its entirety, at one’s own convenience, is invaluable. However, simulation models must be validated to provide reassurance that the model hones those skills for which it was designed. Though further investigation is certainly warranted, our simulation model shows potential as a useful tool for practicing laparoscopic and robot-assisted myomectomy outside the OR.
